# Exploring the Efficacy of Assistive Technology on the Quality of Occupational Performance: a Single-Case Experimental Design Study of the Use of the Activity Chair

**DOI:** 10.1007/s44474-026-00014-2

**Published:** 2026-11-13

**Authors:** Stina Bjørnskov, W. Ben Mortenson, Kirsten Schultz Petersen, Tina Helle

**Affiliations:** 1 Research Centre for Prevention and Rehabilitation, VIA University College, Aarhus, Denmark; 2 Department of Health Science and Technology, Aalborg University, Aalborg, Denmark; 3 Department of Occupational Science and Occupational Therapy, Faculty of Medicine, University of British Columbia, Vancouver, Canada

**Keywords:** Occupational therapy, Activities of daily living, Alternating treatments design, Assistive technology services, Home environment

## Abstract

**Purpose:**

Assistive technology (AT) is provided to support occupational performance, yet evidence on the efficacy of different types of AT is limited. This study aimed to explore the efficacy of the activity chair in relation to the quality of occupational performance.

**Methods:**

This study used an alternating treatments design (ATD) within a single-case experimental design (SCED), involving six first-time users of an activity chair. Across five home visits, data on quality of occupational performance were collected using the Assessment of Motor and Process Skills (AMPS), alongside self-reported measures of effort, efficiency, safety, satisfaction, and independence, as well as data on the time used to complete the AMPS tasks. Data were analysed using visual inspection and statistical comparisons of differences between conditions (with and without the activity chair).

**Results:**

No consistent pattern regarding the efficacy of the activity chair in relation to the quality of occupational performance was identified across participants. One participant demonstrated improved motor skills, whereas the remaining participants showed no clear differences and considerable within- and between-participant variation. Yet, several participants reported lower effort when using the activity chair, while process skills were often lower and task completion time was longer. Findings suggested that the activity chair may facilitate some specific performance skills, such as bending, reaching, lifting, and transporting objects, while making positioning and navigation more challenging.

**Conclusions:**

The findings indicate that the efficacy of the activity chair in relation to the quality of occupational performance may depend on the participant and the specific situation in which it is used. Individualised assessment and training should therefore include observation of relevant occupations in the home environment, with ongoing follow-up to address changing needs over time.

## Introduction

Assistive technology (AT) is needed by an estimated 2.5 billion people worldwide, a number projected to rise to 3.5 billion by 2050 [1]. According to Cook et al., AT refers to items or equipment that are used to increase, maintain, or improve the functional capabilities of individuals with disabilities [2], thereby positioning AT as an intervention that supports the performance of meaningful occupations [3]. On this basis, the provision of AT is justified by the assumption that AT-mediated improvements in occupational performance promote independence, participation, and quality of life [3], outcomes that AT users themselves have identified as high priorities [4, 5]. While different definitions of occupational performance exist within the occupational therapy literature, in this paper occupational performance is understood as the ability to perform meaningful occupations [6], whereas quality of occupational performance refers to how well these occupations are performed in terms of effort, efficiency, safety, and independence [7]. Despite the central role of occupational performance, most research on AT evaluates outcomes either at the level of body functions, such as changes in walking speed when using a walker [8], or in terms of usage factors such as use or non-use, satisfaction, adherence, or acceptance [9–12]. Although some studies have reported improvements in self-reported occupational performance [13–16], these studies remain few and highly heterogeneous in terms of aims, populations, and AT types. Consequently, research evaluating the efficacy of AT in terms of the quality of occupational performance remains scarce [17–19]. This gap is critical in light of the shift in how AT is understood within occupational therapy. AT is no longer viewed merely as compensation for impaired body functions, but as a transactional element that shapes the interaction between the user, the occupation, and the environment, thereby changing occupational demands [7]. It has been argued that AT inherently alters how occupations are performed within unique situations because AT and occupational performance are intertwined [17, 20]. This means that while AT may facilitate certain aspects of occupational performance by reducing some demands, it may simultaneously increase other demands, particularly as challenges often arise in the transaction between the user, the occupation, the AT, and the environment [9, 21–23]. Therefore, it is imperative not only to establish the efficacy of AT in terms of the quality of occupational performance but also to understand how AT alters occupational performance. Understanding both aspects is essential to informing AT provision practice and thereby improving AT outcomes for users and society at large.

Examining the efficacy of AT in terms of the quality of occupational performance requires rigorous evaluation methods, for which randomised controlled trials (RCTs) are traditionally viewed as the most rigorous design [24]. However, the use of RCTs has been problematised in the literature, as methodological features, including standardised intervention protocols, homogeneous samples, and a controlled setting, limit their clinical applicability in AT research [25–27]. In fact, RCT designs contrast with AT research, which typically involves heterogeneous user groups [19] and requires individualised interventions tailored to the user’s unique situation to support occupational performance across different occupations and environments [25–28]. Furthermore, withholding AT from a control group with an identified need for AT is ethically insupportable [27]. Consequently, there is a need for research designs that accommodate such individual differences, while still upholding ecological validity and allowing solid conclusions regarding quality of occupational performance and efficacy of AT [18, 28]. Single-case experimental designs (SCEDs) provide such an alternative as they allow individualised intervention adaptations and can be conducted in the users’ home environments [29, 30] while demonstrating causal efficacy in small and heterogeneous populations [24]. By employing repeated measures of the outcome under different intervention conditions within the same individual, they enable evaluation of intervention efficacy [29], that is, whether systematic changes in quality of occupational performance can be attributed to the use of the AT [31].

Identifying outcome measures of quality of occupational performance that are meaningful for AT is also challenging for two main reasons. First, three recent systematic reviews of outcome measures applied in AT research identified only a few measures related to occupational performance [19, 32, 33], all of which were based on self-report and assessed users’ perceived difficulties with occupational performance before and after an AT intervention using tools such as the Assessment of Life Habits [34], the Canadian Occupational Performance Measure [35] and the Individually Prioritised Problem Assessment [36]. However, these measures focus on perceived occupational performance and do not assess the self-reported quality of occupational performance operationalised in this study as the effort, efficiency, safety, satisfaction and independence with which occupations are performed. Second, since discrepancies across observed and self-reported quality of occupational performance measures are well documented [37–39], a combination of methods is suggested to generate thorough knowledge about the efficacy of AT. Regarding observation-based occupational performance measures, to the best of our knowledge, the Assessment of Motor and Process Skills (AMPS) [40] is among the most well-established assessments for evaluating the quality of occupational performance in a standardised manner. Using the AMPS, it is possible to measure the quality of occupational performance during the performance of standardised tasks at two levels: the overall motor and process skills level and the specific performance skills level [40]. Yet, so far, the AMPS has not been used to evaluate the efficacy of AT.

Following from the transactional understanding of AT [40] and the heterogeneity in the purposes and functions of AT [28, 41], it is evident that a wheelchair, a rollator, or a shower chair alter occupational performance in different ways. Thus, evaluating the efficacy of AT as a generic category is not meaningful [18]. In the present study, we focus on the activity chair as an example of a frequently provided AT [9, 11, 12], with users reporting regular use across diverse occupations in their homes [9]. The activity chair supports seated occupational performance among users experiencing reduced stamina, balance difficulties, or challenges involving bending and reaching [9, 42]. Features such as a seat that is manually or electrically adjustable in height and tilt, a central brake system, and hands-free mobility serve to facilitate the occupational performance of household tasks such as cooking, cleaning, and doing the laundry [42]. Yet, its efficacy remains unknown. Therefore, the aim of this study was to explore the efficacy of the activity chair in relation to the quality of occupational performance. The specific aims were to explore:

A. Differences in the quality of occupational performance, focusing on overall motor and process skills, with and without the activity chair.
B. Differences in the quality of specific performance skills with and without the activity chair.
C. Differences in self-reported quality of occupational performance with and without the activity chair.
D. Differences in the time required to complete tasks with and without the activity chair.

## Material and Methods

### Design

**Fig. 1:**
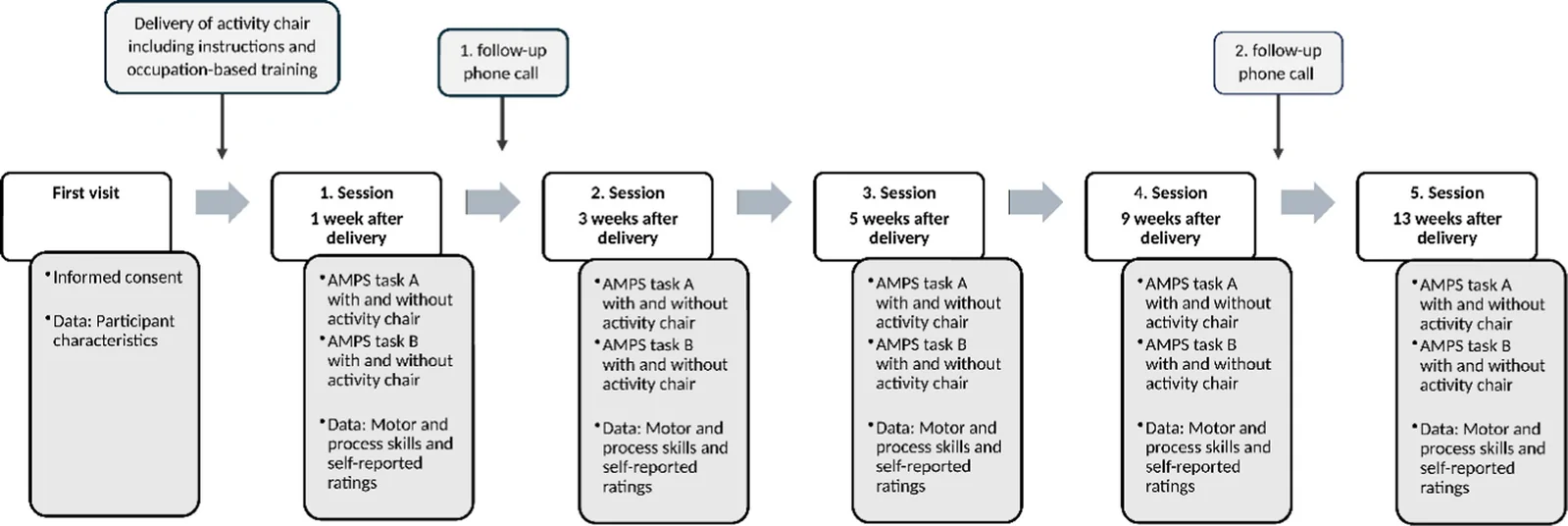
Overview of the intervention and data collection

This is a SCED study using an alternating treatments design (ATD) [29], allowing a within-person comparison of quality of occupational performance in real-life contexts under two conditions: with and without the activity chair. Six activity chair users participated in five sessions each, in which quality of occupational performance was assessed under both conditions with a randomised order (Fig. 1). The study was designed and conducted in accordance with established quality standards for ATDs proposed by the What Works Clearinghouse [43]. The present paper is reported in accordance with the Single-Case Reporting Guideline in Behavioural Interventions (SCRIBE) guideline [44].

### Study Sample

When an activity chair had been granted, occupational therapists providing AT services in four Danish municipalities screened for eligibility based on the following inclusion criteria: (1) granted an electric activity chair for the first time but had not yet received it; (2) aged over 25 years; (3) able to participate in the study; (4) able to speak and read Danish; and (5) living in an ordinary dwelling and not receiving home care. If the criteria were met, the potential participant was informed about the study and invited to participate by the first author. To meet the recommended minimum of three participants for studies using an alternating treatments design [43], recruitment targeted six participants to allow for potential dropout. Recruitment using this purposive sampling technique [45] resulted in a sample of six female participants with a mean age of 57 years (Table 1). They had varying functional limitations and used a combination of different mobility devices. Four participants were retired, one was on sick leave, and one was in subsidised employment.

**Table 1:** Participant characteristics of the *n* = 6 female participants

Participant	Age	Living arrangement	Functional limitations^a^	Use of mobility devices^a^	Employment status
1	75 years	Lived alone	Limitations of stamina, reduced spine and/or lower extremity function	Uses elbow crutches indoors and outdoors	Retired
2	26 years	Lived alone	Poor balance, incoordination, limitations of stamina, reduced function in upper extremities, reduced fine motor skills, reduced spine and/or lower extremity function	Uses one elbow crutch outdoors	On sick leave
3	69 years	Lived alone	Poor balance, Limitations of stamina, reduced spine and/or lower extremity function	Uses a walker indoors and outdoors and a wheelchair outdoors	Retired
4	63 years	Lived alone	Poor balance, Incoordination, limitations of stamina, difficulty moving head, reduced function in upper extremities, reduced fine motor skills, reduced spine and/or lower extremity function	Uses a high walker indoors and outdoors	Early retirement
5	51 years	Lived with spouse	Poor balance, incoordination, limitations of stamina, difficulty moving head, reduced function in upper extremities, reduced spine and/or lower extremity function	Uses cane indoors and outdoors, and wheelchair outdoors	Subsidised employment
6	59 years	Lived with spouse	Poor balance, incoordination, limitations of stamina, difficulty moving head, reduced function in upper extremities, reduced fine motor skills, reduced spine and/or lower extremity function	None	Early retirement

^a^Functional limitations and use of mobility devices were assessed by means of the Person Component of the Housing Enabler [46]

### Intervention and Control Condition

The activity chair intervention consisted of the provision of an individually adjusted activity chair, thorough instructions about adjustment options and occupation-based training in the participant’s home as well as two follow-up phone calls and, if needed, additional home visits and training to support the participants’ use of the activity chair. The activity chair (VELA Tango 700E, ISO 9999 classification 18 09 03 12) was equipped with electric height adjustment, seat tilt, a central brake system, and small wheels allowing hands-free mobility while seated. The intervention was carried out by an experienced activity chair specialist. To support procedural fidelity, the specialist followed a checklist covering the core components of the intervention, while the delivery of these components was tailored to each participant.

In this study, which followed an ATD, quality of occupational performance was assessed by comparing performance of the same AMPS tasks with and without the activity chair. In the intervention condition (with), participants were instructed to use their individually adjusted activity chair during all parts of the task where its use was possible and appropriate. In the control condition (without), participants were instructed to perform the task as they did prior to receiving the activity chair, for example, by using a dining chair or walker, or asking for assistance. Thus, participants could use different ways of performing the tasks in the control condition, depending on the task and their individual needs, rather than following a standardised procedure.

### Outcomes and Data Collection

Observed quality of occupational performance was assessed using the AMPS [40], which provides measures of motor skills (16 items) and process skills (20 items) reflecting the quality of occupational performance during standardised AMPS tasks. Motor skills reflect the physical effort involved, whereas process skills reflect task organisation and efficiency [40]. The AMPS yields overall motor and process skills scores reported in logits based on the performance of two standardised AMPS tasks, with higher scores indicating better quality of occupational performance. A difference of ≥ 0.5 logits is considered clinically meaningful [40]. In addition, all 36 items representing specific performance skills are rated using a 4-point ordinal scale. The AMPS can be repeated with minimal retest effects and has excellent psychometric properties for the overall motor and process measures [40].

Informed by the AMPS parameters [40], a study-specific assessment consisting of five questions was developed to measure self-reported quality of occupational performance using an 11-point numerical rating scale. The questions addressed (1) effort: perceived fatigue, shortness of breath, or feelings of clumsiness, (2) efficiency: perceived time use, task pacing, and whether parts of the task had to be redone, (3) safety: perceived risk of personal injury or damage to objects or the surrounding environment, (4) satisfaction with task performance, and (5) independence: the need for assistance from others. The questions and rating scale were critically reviewed by three individuals representing the target sample of this study to examine face validity [47].

In addition, the duration of each of the AMPS tasks performed [40] was recorded using a stopwatch.

At each session, the participant performed two standardised AMPS tasks that were observed and rated by the same trained and calibrated AMPS rater (an occupational therapist) [40], under both conditions (with and without the activity chair) in a randomised order. For each AMPS task, the participant first performed the task either with or without the activity chair, followed by performance of the same task under the opposite condition. The same procedure was repeated for the second AMPS task. After completion of each AMPS task, the participant answered the five questions about perceived efficiency, effort, safety, satisfaction, and independence.

In accordance with the AMPS, tasks were selected collaboratively by the participant and the occupational therapist prior to each session to reflect occupations that the participant would realistically perform in a real-life context [40]. Tasks were selected to represent occupations for which the activity chair was intended to support occupational performance and could vary between sessions to maintain relevance across the study period [40]. Selection was further guided by safety considerations, and AMPS tasks that could not be performed safely with and/or without the activity chair were excluded. Neither the participants nor the occupational therapist conducting the data collection could be blinded due to the nature of the study procedures. No procedural changes or adverse events occurred during the study.

To strengthen internal validity in the ATD, the order of conditions (with or without the activity chair) was randomly assigned for each session [48]. Randomisation was performed using the SLUMP function in Microsoft Excel [49] by a research assistant who was not involved in the study. As each session involved two AMPS tasks, each performed with and without the activity chair, the order of conditions could follow one of four possible sequences (ABAB, BABA, ABBA, or BAAB). Table 2 presents the randomisation sequence for each participant across the five sessions, showing the order of the two performance conditions, without (A) and with (B) the activity chair.

**Table 2:** Randomisation sequence for each of the *n* = 6 participants

Participant	Randomisation sequence
1	BAAB BAAB BAAB BABA ABBA
2	ABAB BAAB BABA BAAB ABBA
3	ABAB ABBA BAAB ABBA BABA
4	BAAB BABA ABAB BAAB ABBA
5	BABA ABAB BABA ABBA ABBA
6	ABBA ABAB BAAB ABBA BAAB

### Data Analysis

Data analyses were led by the first author, who is trained and calibrated in the use of AMPS. Following recommendations for analyses of ATD data, visual and statistical analyses were applied [50]. The significance level was set at α = 0.05. Data were organised in Microsoft Excel [49]. Statistical analyses and graphical outputs were generated in R [51] using RStudio [52].

For each participant, line graphs were created for overall motor and process skills. Visual analysis was conducted for each participant to explore patterns in level, trend, and magnitude of differences in quality of occupational performance with and without the activity chair. To quantify the between-condition difference in AMPS scores across repeated observations, the Average Difference between Successive Observations (ADISO) [53] was calculated for each participant with corresponding 95% confidence intervals. In this study, each session included one AMPS score per condition (one with and one without the activity chair), making ADISO equivalent to the mean difference across the five sessions. Statistical significance of the ADISO estimates was assessed using a randomisation test (Monte Carlo permutation, 10,000 iterations) [54]. An exact Wilcoxon signed-rank test was conducted as a supplementary, exploratory analysis to examine the robustness of the findings [55].

For each of the 36 specific performance skills, skill ratings were paired within each participant by matching ratings obtained during the same AMPS task performed with and without the activity chair. For each skill, each participant generated 20 skill ratings (10 with and 10 without the activity chair), resulting in 10 paired skill ratings per participant. For each pair, we determined whether the skill rating was higher when the AMPS task was performed with the activity chair, higher when performed without the activity chair, or equal across the two conditions. Descriptive statistics were calculated as percentages of pairs in which the skill rating was higher with the activity chair, higher without the activity chair, or equal across the two conditions (ties). McNemar’s test [56] was applied to the discordant pairs to examine whether skill ratings were more frequently higher in one condition than the other, with ties excluded. The 60 pairs were derived from only six participants; therefore, the assumption of independence underlying McNemar’s test [56] was not fully met. The results should therefore be interpreted as exploratory. To further explore whether the observed differences were driven by specific participants, a complementary descriptive analysis was conducted. For each participant and each skill, we summarised the number of occasions (out of 10 pairs) in which the skill rating was higher when the AMPS task was performed with versus without the activity chair. The results are presented in bar charts showing the distribution for each skill.

Regarding self-reported effort, efficiency, safety, and satisfaction, ratings from the two AMPS tasks performed with the activity chair and the two performed without it within the same session were averaged separately for each outcome. Line graphs were created for each participant, and differences between the averages were visually inspected.

For each participant, mean AMPS task time was calculated separately for tasks performed with and without the activity chair across all sessions. Participant-level differences were summarised as the mean difference, median difference, and standard deviation.

### Procedures

Data were collected in the participants’ home from May 2024 to August 2025. At the first home visit, the first author provided additional information about the study purpose and procedures and collected data on participant characteristics. The activity chair was then delivered by the specialist, who also provided instructions and tailored occupation-based training. Following delivery, the occupational therapist conducting the data collection contacted the participant to agree on two AMPS tasks to be performed at the first session. Data were subsequently collected by the same occupational therapist during five sessions at 1, 3, 5, 9, and 13 weeks after delivery of the activity chair to meet standards of at least five data points per condition [43]. Sessions were scheduled more closely during the initial weeks to capture early changes in quality of occupational performance, while the longer intervals between later sessions allowed participants more time to become familiar with using the activity chair. Session duration varied but was typically around 3–4 h, with breaks provided according to the participants’ needs. Between the sessions, the occupational therapist and participant agreed on the AMPS tasks to be performed at the following session. The activity chair specialist conducted two follow-up phone calls 2 and 9 weeks after delivery.

## Results

The selected AMPS tasks included meal preparation, cleaning, laundry, table setting, and other household occupations, with difficulty levels ranging from much easier to much harder than average ADL tasks [40]. Some participants repeatedly selected the same tasks, while others changed tasks several times during the study period. No consistent pattern in task difficulty over time was identified.

### Differences in the Overall Quality of Occupational Performance

**Fig. 2:**
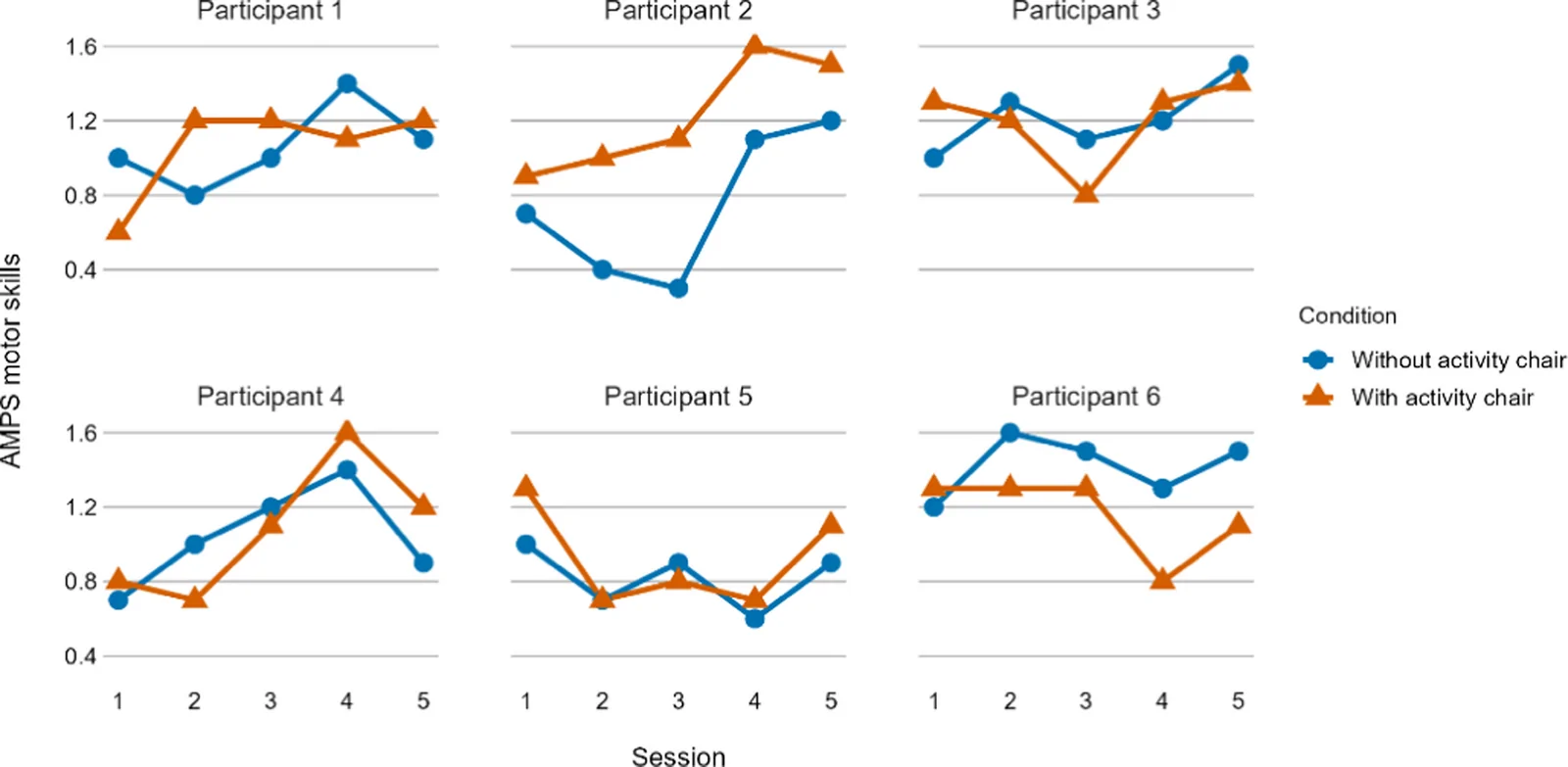
AMPS motor skills across sessions with and without the activity chair

Differences in observed motor skills showed overlapping patterns for four of the six participants, with variability across sessions and an inconsistent pattern between conditions (Fig. 2). Only Participant 2 demonstrated consistently higher motor skills across sessions when performing the AMPS tasks with the activity chair, with scores showing an upward trend over time, whereas Participant 6 showed higher motor skills in four of the five sessions without the activity chair.

**Fig. 3:**
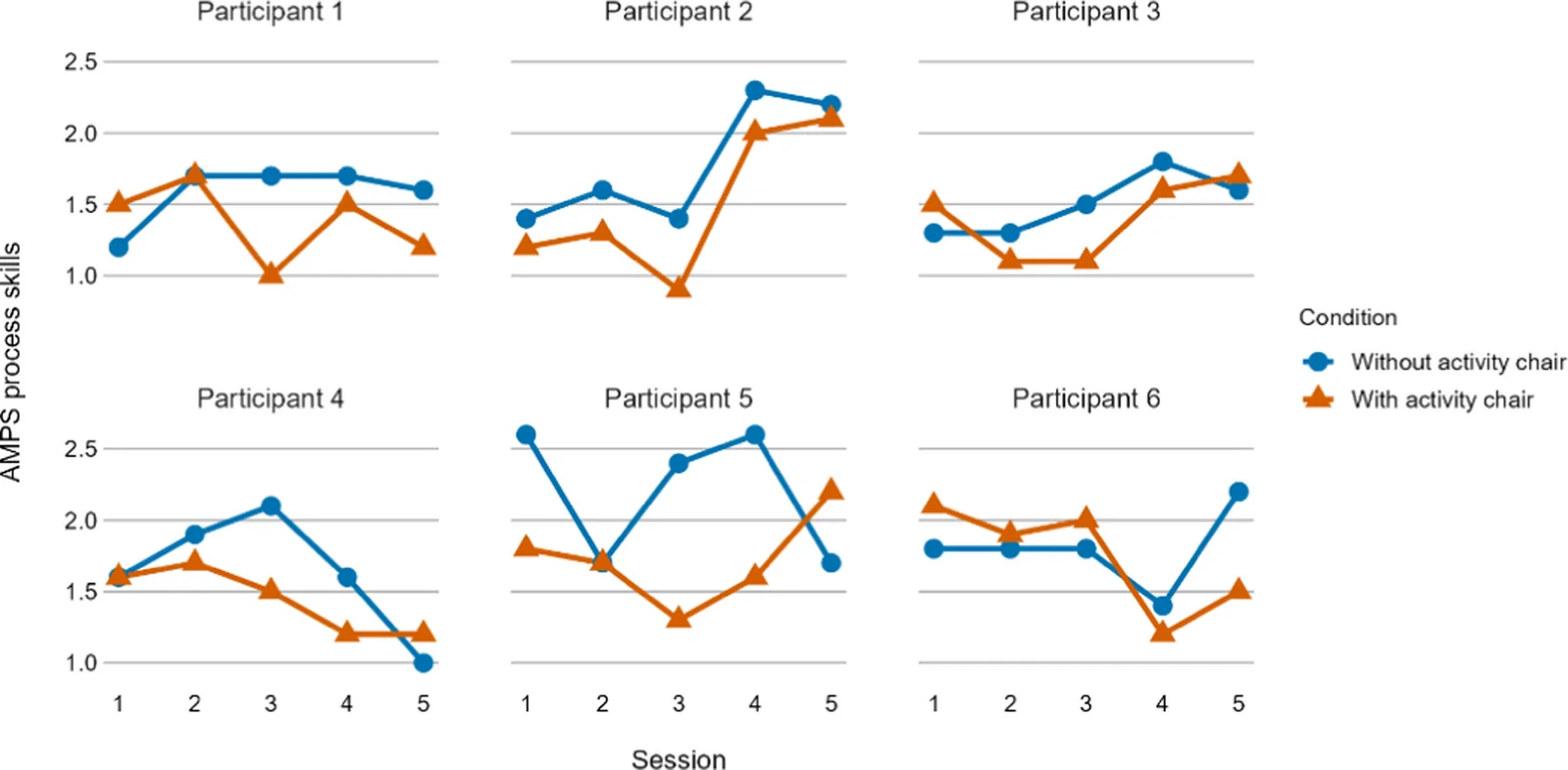
AMPS process skills across sessions with and without the activity chair

Regarding differences in overall process skills across the five sessions, scores were lower when tasks were performed with the activity chair for four of the six participants, although some variability was observed (Fig. 3). Participant 2 demonstrated consistently lower process skills with the activity chair across sessions. Participants 1, 3, and 4 also demonstrated lower process skills with the activity chair and minimal differences between conditions. In contrast, Participant 5 demonstrated variability in process skill scores across sessions, with fluctuating differences between conditions and no consistent pattern.

Regarding the average differences in overall successive motor and process skills across sessions, the ADISO estimates indicate only small between-condition differences for most participants (Table 3). For motor skills, Participant 2 showed a tendency toward higher scores when AMPS tasks were performed with the activity chair. For process skills, all participants showed higher scores when AMPS tasks were performed without the activity chair. However, differences in process skills were not statistically significant and varied in magnitude across participants.

**Table 3:** Differences in motor and process skills (logits) during AMPS task performance with and without the activity chair

Participant	AMPS motor skills				AMPS process skills			
	Adiso^a^	95% CI	Randomisation *p*-value^b^	Exact *p* value^c^	Adiso^a^	95% CI	Randomisation *p* value^b^	Exact *p* value^c^
1	0.00	(− 0.42;0.42)	1.00	1.00	− 0.20	(− 0.53; 0.13)	0.833	0.273
2	0.48	(0.18;0.78)	0.059	0.063	− 0.28	(− 0.41; 0.15)	0.650	0.633
3	− 0.02	(− 0.30;0.26)	1.000	1.000	− 0.10	(− 0.32; 0.12)	0.267	0.438
4	0.04	(− 0.26;0.34)	0.683	0.813	− 0.20	(− 0.48; 0.08)	0.167	0.198
5	0.10	(− 0.10;0.29)	0.374	0.375	− 0.48	(− 1.09; 0.13)	1.000	0.144
6	− 0.22	(− 0.62,0.17)	0.256	0.250	− 0.06	(− 0.41; 0.29)	0.983	1.000

^a^Average difference between successive observations [53]: positive values correspond to higher skills with the activity chair

^b^Randomisation test [54]

^c^Exact Wilcoxon signed-rank test [55]

### Differences in the Quality of Specific Performance Skills

McNemar’s test indicated statistically significant differences for seven specific performance skills when comparing paired skill ratings for AMPS tasks performed with and without the activity chair (Table 4). Among the motor skills, *lifts*, *transports*, *reaches*, and *bends* were more often rated higher with the activity chair, whereas *moves* and *positions* were more often rated higher without it. Among the process skills, *navigates* was more often rated higher without the activity chair.

**Table 4:** Skill-level distribution of paired AMPS observations showing higher scores when AMPS tasks were performed with versus without the activity chair across participants

	Higher score with activity chair	Higher score without activity chair	McNemar *p* value^a^
	Count (%)	Count (%)	
Motor skills			
Endures	9 (15.0)	8 (13.3)	1.000
Aligns	17 (28.3)	8 (13.3)	0.110
Lifts	15 (25.0)	1 (1.7)	0.001*
Moves	2 (3.3)	20 (33.3)	0.001*
Transports	21 (35.0)	4 (6.7)	0.001*
Flows	16 (26.7)	10 (16.7)	0.327
Grips	10 (16.7)	13 (21.7)	0.677
Reaches	14 (23.3)	3 (5.0)	0.016*
Walks	0 (0.0)	4 (6.7)	0.134
Bends	14 (23.3)	3 (5.0)	0.015*
Manipulates	4 (6.7)	1 (1.7)	0.371
Stabilises	7 (11.7)	4 (6.7)	0.546
Coordinates	8 (13.3)	9 (15.0)	1.000
Paces	6 (10.0)	16 (26.7)	0.055
Calibrates	6 (10.0)	11 (18.3)	0.332
Positions	5 (8.3)	19 (31.7)	0.008*
Process skills			
Uses	4 (6.7)	0 (0.0)	0.134
Chooses	6 (10.0)	8 (13.3)	0.789
Sequences	1 (1.7)	6 (10.0)	0.131
Searches/Locates	4 (6.7)	4 (6.7)	0.724
Attends	2 (3.3)	6 (10.0)	0.289
Inquires	5 (8.3)	9 (15.0)	0.423
Heeds	2 (3.3)	9 (15.0)	0.070
Gathers	7 (11.7)	4 (6.7)	0.546
Terminates	5 (8.3)	9 (15.0)	0.423
Adjusts	8 (13.3)	11 (18.3)	0.646
Navigates	7 (11.7)	26 (43.3)	0.002*
Handles	4 (6.7)	13 (21.7)	0.052
Restores	8 (13.3)	8 (13.3)	0.803
Initiates	7 (11.7)	7 (11.7)	0.789
Continues	13 (21.7)	13 (21.7)	0.845
Organises	6 (10.0)	12 (20.0)	0.789
Notices/Responds	6 (10.0)	8 (13.3)	0.646
Benefits	8 (13.3)	11 (18.3)	0.248
Accommodates	3 (5.0)	0 (0.0)	NA

^a^McNemar’s test [56]

*Statistically significant difference between conditions (*p* < 0.05)

**Fig. 4:**
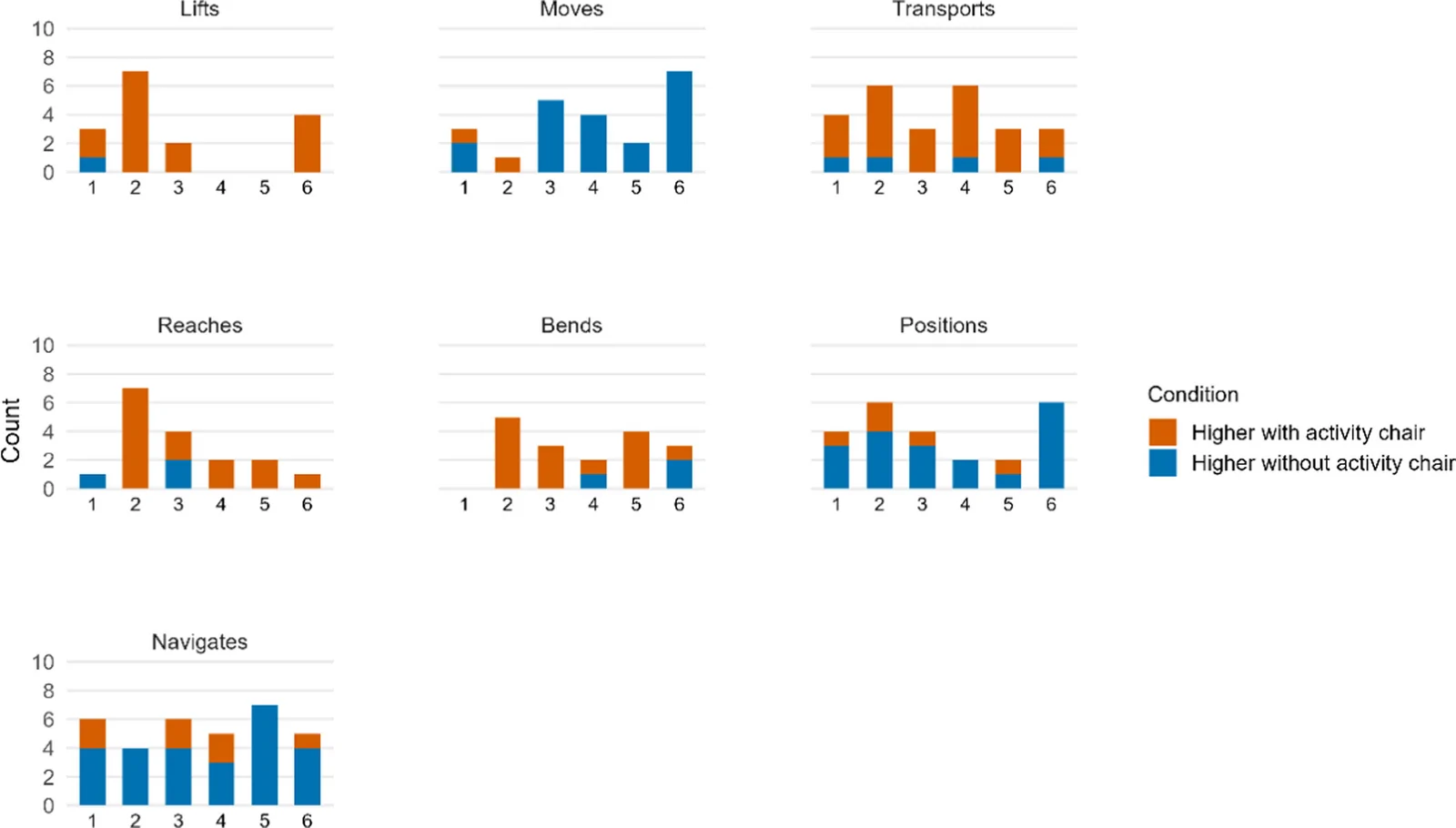
Participant-level distribution of paired skill ratings for the seven specific performance skills that showed statistically significant differences in Table 4

The pattern of paired skill ratings for the seven specific performance skills varied across participants (Fig. 4). However, for several skills, higher ratings in one condition were distributed across most or all participants. For example, across all participants, *transports* was more often rated higher when AMPS tasks were performed with the activity chair, and *navigates* was more often rated higher without it. In contrast, for *lifts* and *bends*, higher ratings with the activity chair were primarily observed in a few participants, while others showed little difference between conditions or higher ratings without it.

### Differences in Self-reported Quality of Occupational Performance

**Fig. 5:**
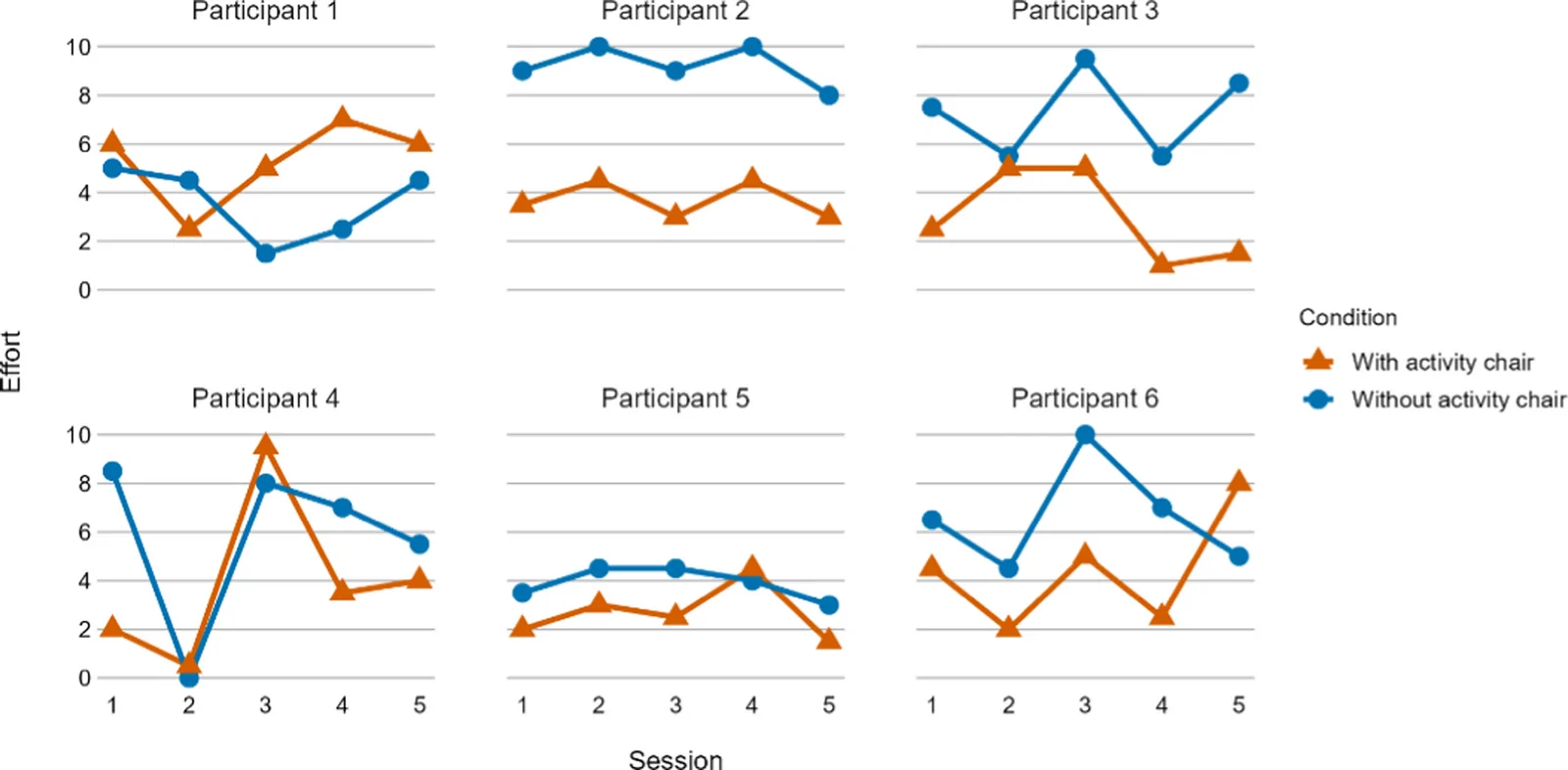
Self-reported effort during AMPS task performance with and without the activity chair, rated on a 0–10 scale. Higher scores indicate greater perceived effort

Four participants (2, 3, 5, and 6) generally reported lower effort when using the activity chair, indicating reduced perceived physical demand (Fig. 5). However, individual ratings sometimes overlapped or were very close between conditions. In contrast, Participants 1 and 4 showed no clear pattern of differences in self-reported effort between conditions.

**Fig. 6:**
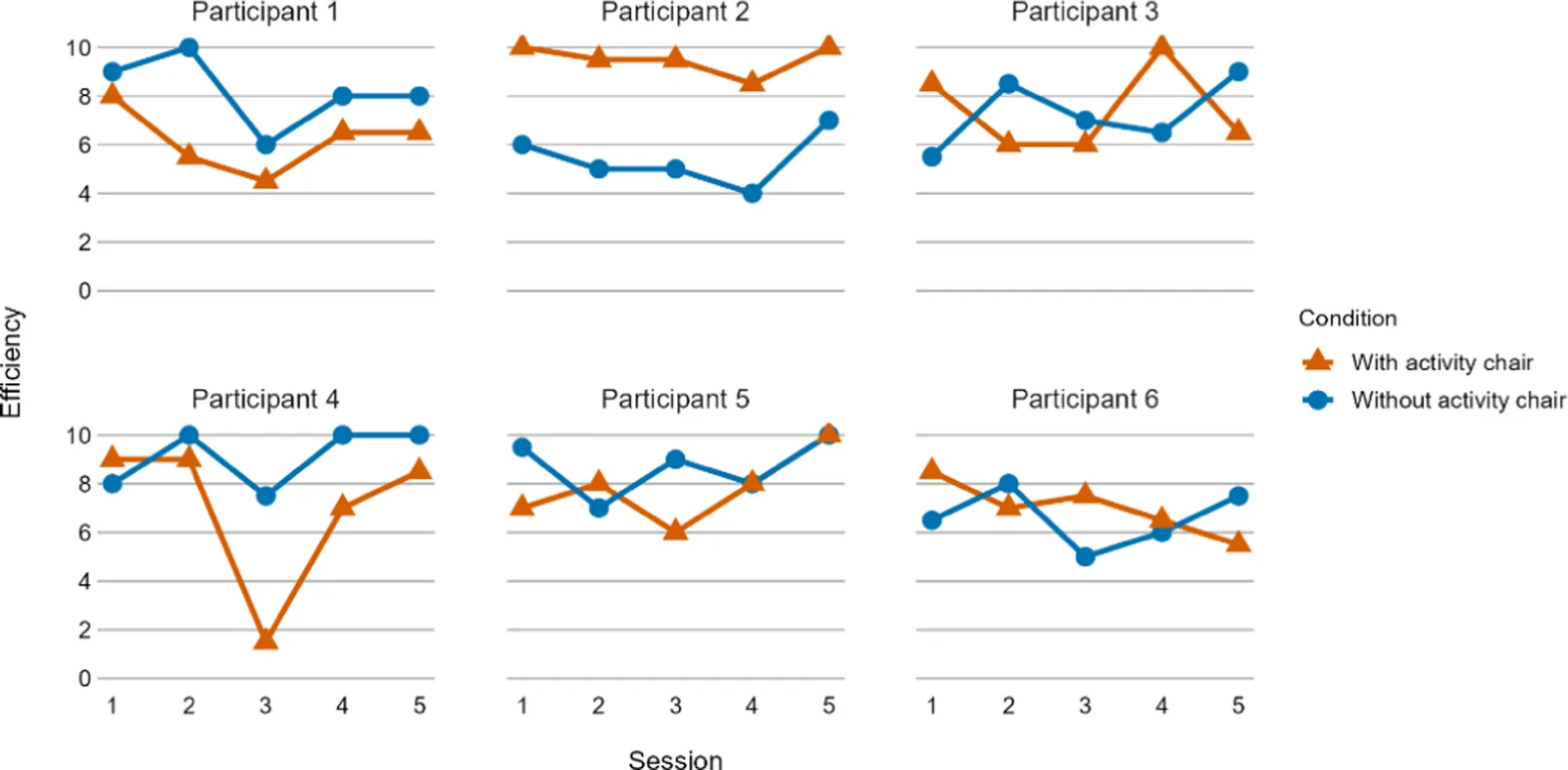
Self-reported efficiency during AMPS task performance with and without the activity chair, rated on a 0–10 scale. Higher scores indicate greater perceived efficiency

The pattern of self-reported efficiency varied across participants (Fig. 6). Participant 2 consistently reported higher efficiency when using the activity chair, whereas Participant 1 consistently reported higher efficiency without the chair. For Participants 3 and 4, self-reported efficiency was generally higher without the activity chair. Participants 5 and 6 showed inconsistent patterns, with self-reported efficiency fluctuating between sessions.

**Fig. 7:**
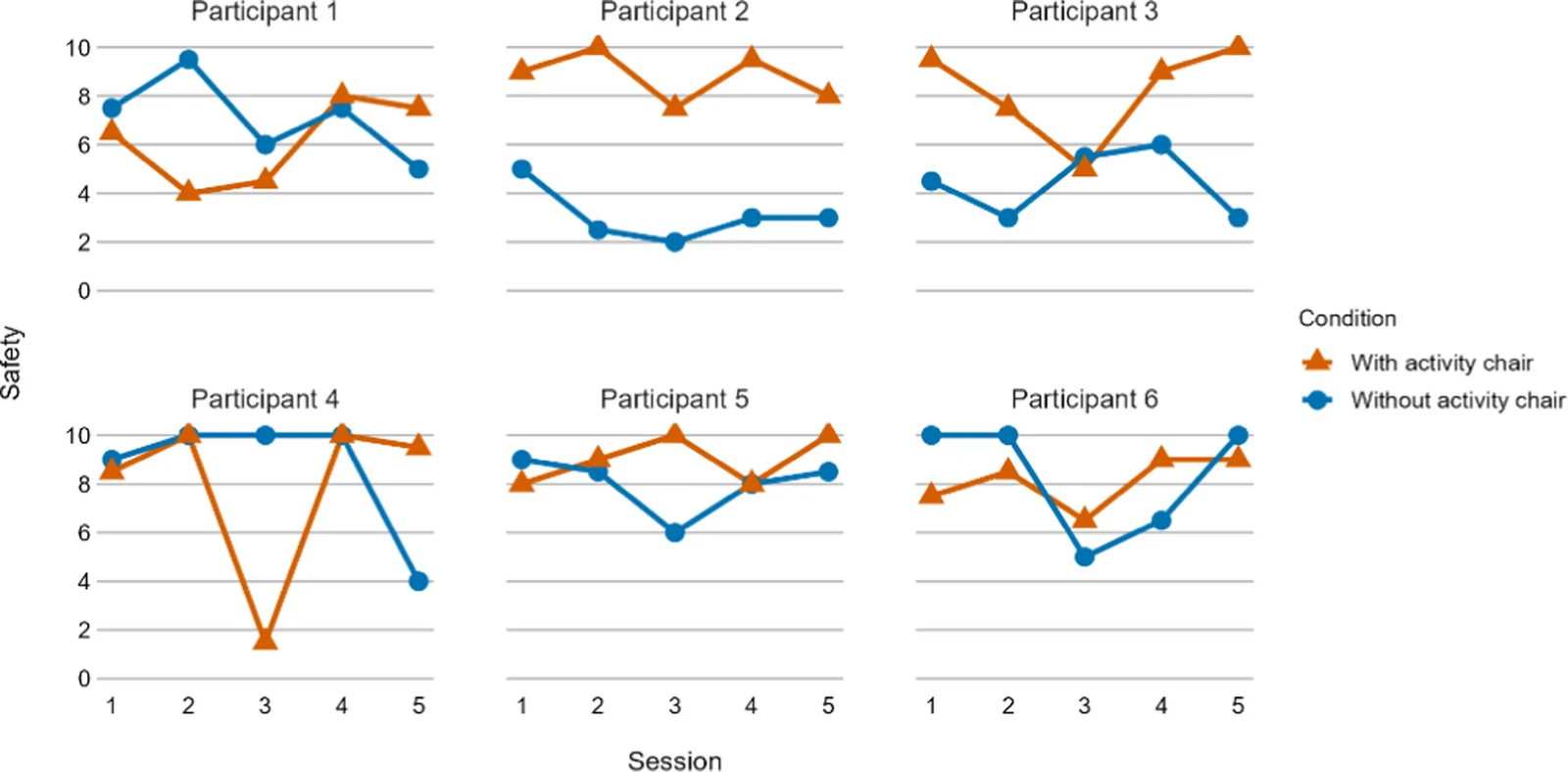
Self-reported safety during AMPS task performance with and without the activity chair, rated on a 0–10 scale. Higher scores indicate greater perceived safety

As for self-reported safety, Participants 2 and 3 generally reported higher safety when AMPS tasks were performed with the activity chair compared with when performed without the chair (Fig. 7). Apart from a single exception for Participant 3, both participants showed higher self-reported safety when using the activity chair. For the remaining four participants, the pattern was more inconsistent, with varying differences in safety between conditions.

**Fig. 8:**
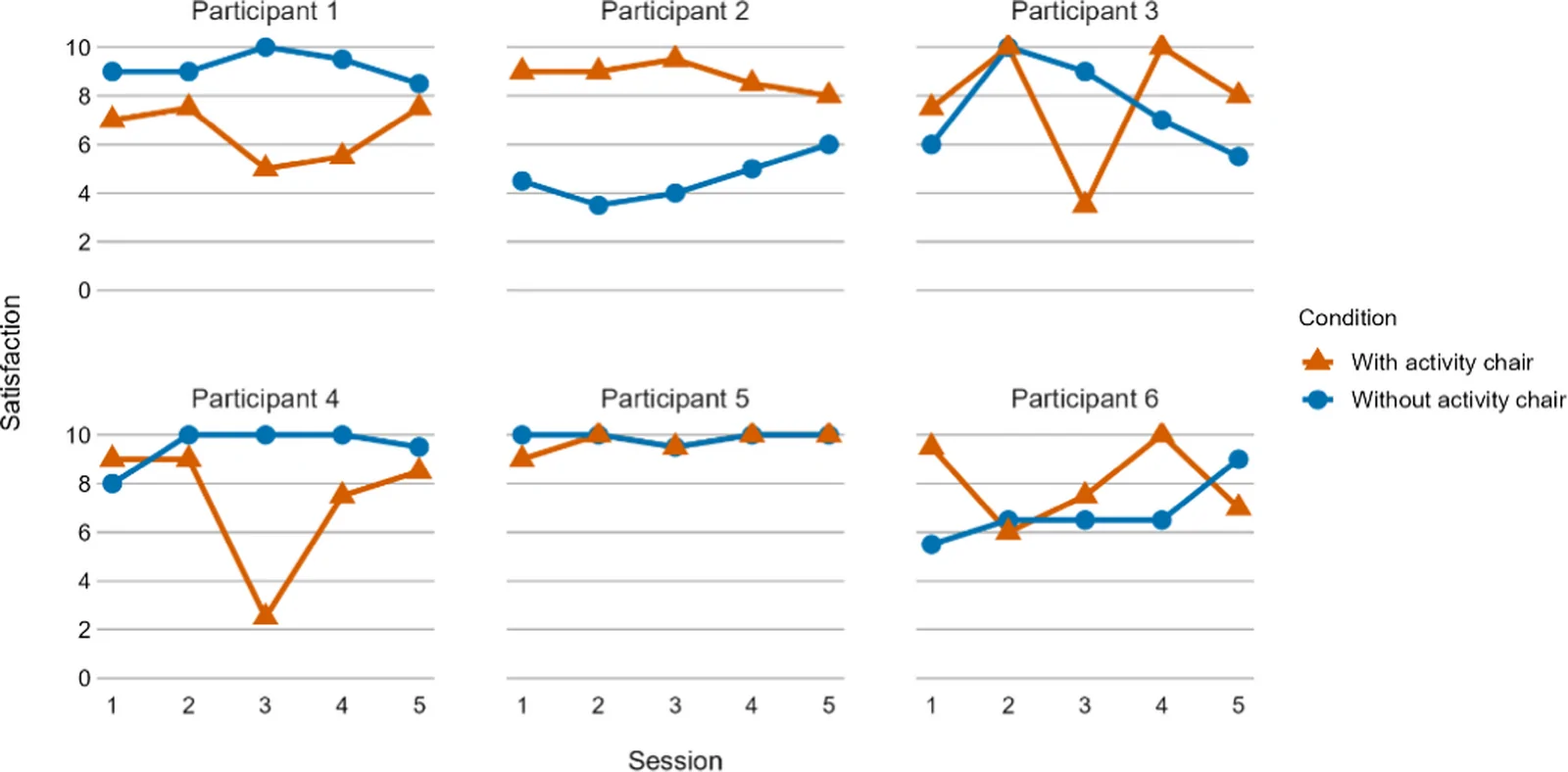
Self-reported satisfaction with AMPS task performance with and without the activity chair, rated on a 0–10 scale. Higher scores indicate greater satisfaction

Regarding self-reported satisfaction, the pattern was mixed as well (Fig. 8). Participant 2 consistently reported higher satisfaction when AMPS tasks were performed with the activity chair, whereas Participants 1 and 4 reported higher satisfaction without the chair. In addition, Participants 1, 4, and 5 consistently reported high satisfaction when AMPS tasks were performed without the activity chair, with ratings at or close to the upper end of the 0–10 scale across sessions.

**Fig. 9:**
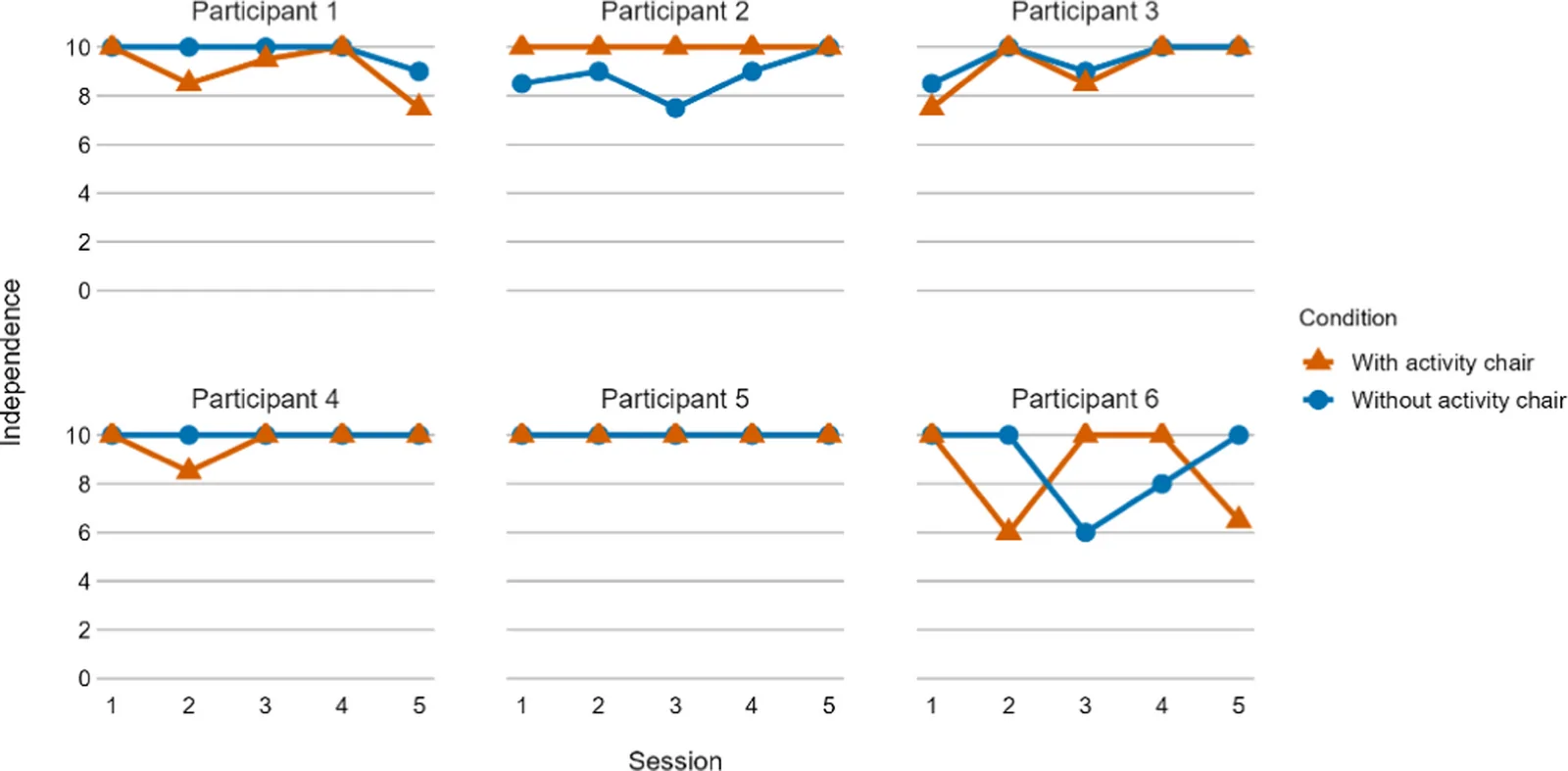
Self-reported independence in AMPS task performance with and without the activity chair, rated on a 0–10 scale. Higher scores indicate greater independence

For self-reported independence, ratings were generally high across participants and sessions (Fig. 9). Participants 2, 3, 4, and 5 reported independence ratings at or close to the upper end of the 0–10 scale when AMPS tasks were performed both with and without the activity chair. Participants 1 and 6 showed more variation across sessions and conditions.

### Differences in the Time Required to Complete Tasks

Concerning mean AMPS task time, five out of six participants required approximately 1 to 2.5 min longer to complete the tasks when using the activity chair, with Participant 1 showing the largest increase (Table 5). Only Participant 2 completed the tasks faster with the activity chair, although the difference was less than 0.2 min.

**Table 5:** Participant-level differences in mean AMPS task time in seconds (s) with and without the activity chair

Participant	Mean time without the activity chair (s)	Mean time with the activity chair (s)	Mean difference (s)	Median difference (s)	Standard deviation of differences (s)
1^a^	345	492	147	127	156.37
2	336	325	− 11	0	39.00
3	263	327	64	45	46.63
4	306	410	104	102	55.33
5	242	340	98	82	42.13
6	261	381	120	132	68.69

^a^AMPS task completion time was not recorded for Participant 1 during the first two sessions

## Discussion

In this study, we explored the efficacy of the activity chair in improving the quality of occupational performance using observed and self-reported measures. For observed overall motor skills, a clear improvement was identified for one participant, whereas the remaining five participants demonstrated overlapping patterns across conditions, with considerable between-participant variability and within-participant fluctuations. This may reflect what previous research has suggested, namely that outcomes of AT are highly individual and context-dependent [17, 21, 28]. In contrast, four participants generally reported lower effort when performing tasks with the activity chair, indicating reduced perceived physical demand, although ratings sometimes overlapped or were very close between conditions. This finding further supports previous research showing that observed and self-reported quality of occupational performance represent related but distinct aspects and may not necessarily align [37–39].

Consistent with the theoretical perspective that AT alters the demands of occupational performance [7], the present study found that the activity chair altered the demands placed on specific performance skills. Participants tended to benefit from using the activity chair, particularly when performing tasks involving forward bending and reaching for, lifting, and transporting objects. Yet, using the activity chair also introduced new challenges, especially related to the interaction with the environment, as the activity chair or its brake frequently came into contact with cabinets, doorframes, or other environmental obstacles. This is a well-known issue associated with the use of the activity chair [9] and is in line with broader research demonstrating how environmental barriers impact and constrain AT use [21]. Finally, using the activity chair generally increased task completion time, likely because its use introduced additional steps and demands, such as positioning, adjusting, and operating the chair within the task. This is a well-documented phenomenon in AT research, where AT may require extra time and effort to use [10, 58, 59]. Overall, these findings support existing research suggesting that AT may simultaneously address certain challenges while introducing new constraints to occupational performance [23].

Given the variation in outcomes across participants and tasks observed in the present study, the results support a transactional understanding of AT, where efficacy emerges through the dynamic interaction between the individual, the occupation and its demands, the AT, and the environment [7]. Furthermore, the activity chair altered occupational demands differently across specific tasks, showing how AT and occupational performance are inherently intertwined [20]. Together, these findings imply that the efficacy of AT, such as the activity chair, cannot be isolated from the context and should therefore be understood and evaluated in specific situations [17]. Accordingly, when evaluating efficacy in terms of quality of occupational performance, what is captured is not the AT in itself, but the quality of the transaction in which it is used. This also suggests that variation in efficacy may reflect differences in how well the AT fits the user and the unique situation, which in turn is dependent on the quality of the provision process [60, 61]. This is consistent with previous research demonstrating that the provision process influences outcomes such as use and satisfaction [10, 21, 62, 63]. From this perspective, the provision process is essential for interpreting the findings of the present study, as the activity chair had been granted through routine municipal procedures beyond the control of the study. During data collection, it was informally noted that several participants reported having been granted the activity chair following telephone consultations without a home visit or an occupation-based assessment. In addition, two participants chose to return the activity chair after the study period because it did not fit their needs. This may contribute to explaining why the activity chair did not lead to consistent improvements in quality of occupational performance, and may highlight that the efficacy of AT on quality of occupational performance is closely linked to the provision process and cannot be examined in isolation from how the AT is provided [28].

### Clinical Implications

As suggested by the findings, identifying the right match between the person and the AT is complex. It requires not only professional analytical skills and knowledge of how AT transacts with the user and the environment during occupational performance [7, 61] but also a structured provision process to support the identification of an optimal match [62, 63]. Given the transactional relationship between the user, the AT, the occupation, and the environment, it is important that assessment and training take place in relevant environments, a point also raised in earlier studies [59, 64]. Regarding the activity chair, this study indicates that individuals who experience difficulties with forward bending, reaching, and lifting or transporting objects, and who report increased effort during occupational performance, represent the target population for this AT. It is commonly used during meals and for household tasks such as cooking, laundry, and cleaning [9]. However, clinicians should be aware that the activity chair may simultaneously shift demands to other aspects, such as those involving positioning, manoeuvring, and adjusting the activity chair within the environment. This further highlights the importance of assessment and training in the provision process, as also emphasised in previous research [9, 62, 63]. As the activity chair is used across multiple occupations [9] and may influence occupational performance differently depending on the occupation, we propose that clinicians assess and train the use of the activity chair across a range of relevant occupations. During assessment, particular attention should be paid to the interaction between the activity chair and the environment, as environmental barriers may constrain use and introduce additional challenges [9, 21, 22]. In practice, however, a previous study found that many users merely received the activity chair without further instructions, and only a minority obtained occupation-based training, while 65% of users reported environmental barriers such as lack of space, doorsteps and elevation differences [9]. Considered alongside the present findings, these results reinforce the importance of observing the person in their own environment to identify environmental barriers and potential needs for adaptations, thereby supporting a more optimal fit between the person and the AT in the occupational context. Finally, as users have been found to integrate the activity chair into new occupations over time [9], follow-up is essential to identify emerging challenges, support appropriate use, and reduce potential risks [9]. Given that insufficient follow-up is common in current practice [22], this underscores the importance of understanding AT provision as an ongoing process rather than a discrete event [62, 63].

### Methodological Considerations

Interpreting changes in the quality of occupational performance in the present study requires consideration of key methodological aspects. First, the AMPS requires participants to perform two tasks of sufficient difficulty to generate motor and process skills scores [40]. Following the ATD design [29], each participant performed the same tasks both with and without the activity chair in each session, resulting in four task performances per session. For safety reasons and to avoid excessive fatigue, task selection was therefore limited, which may have lowered task difficulty and thereby reduced sensitivity to detect between-condition differences in the quality of occupational performance [40]. This is supported by the overall high self-reported independence across participants, indicating that the selected tasks may not have required the activity chair for successful completion. High independence was particularly evident for Participants 1, 4, and 5, who also reported consistently high levels of perceived satisfaction when performing tasks without the activity chair, indicating limited room for measurable improvement and a potential ceiling effect on the self-reported outcomes [65]. In contrast, Participant 2 reported lower independence and satisfaction without the activity chair and consistently performed better across both observed motor skills and self-reported outcomes when using the activity chair. This aligns with previous research suggesting that the benefits of AT may be most evident when it enables occupations that would otherwise be difficult or impossible to perform [66]. These considerations reflect an inherent trade-off in AT research conducted in real-life contexts, where ecological validity and safety may come at the expense of experimental control and sensitivity in detecting between-condition differences [25, 26]. Balancing relevance, safety, and task challenge is therefore a critical methodological issue.

Second, given that relevant tasks may evolve over time, and are therefore difficult to predetermine [9], participants were asked to select AMPS tasks of personal relevance involving the activity chair prior to each session to increase ecological validity [67]. While the AMPS is designed to provide valid measures across different tasks [40], the use of different tasks across sessions means that the activity chair was applied in varying occupational contexts. As the activity chair alters occupational demands differently depending on the task, this may have contributed to the within-participant variability in the quality of occupational performance. Furthermore, this may also help explain why no clear increase in the quality of occupational performance over time was observed when using the activity chair, as variations in task demands across sessions may have outweighed potential effects of increased familiarity described in the AT literature [68].

Our findings add to the evidence supporting the value of combining observation and self-report to achieve complementary information [37–39]. In AT research, it is well established that self-reported outcomes are essential, as continued use is less likely when AT is not perceived as useful [10, 69]. Accordingly, perceived improvements, such as reduced effort, may represent important outcomes, even in the absence of observable changes in the quality of occupational performance. However, it is relevant to note that improvements in quality of occupational performance do not necessarily translate into sustained use of AT, as psychological and contextual factors alone may lead to non-use [10]. Conversely, commonly applied outcomes in AT research, such as use, satisfaction, or acceptance [10, 22, 62, 63, 70, 71], do not in themselves indicate whether AT supports occupational performance or enhances independence. This reflects a broader challenge in AT research, where outcome measures have been described as conceptually fragmented, with commonly used constructs applied inconsistently [32, 72]. Taken together, these findings suggest that capturing the impact of AT may require consideration of multiple outcomes, without a clear consensus on how these outcomes should be defined or which outcomes should be prioritised [28].

### Strengths and Limitations

A key strength of this study is the adherence to established standards for conducting SCEDs [43], including an adequate sample size, sufficient repeated measurements, and randomisation of conditions to mitigate potential learning and fatigue effects [48]. A further strength is the use of the AMPS, a standardised and well-established measure of quality of occupational performance with documented validity and sensitivity to change [40]. Procedural fidelity was further supported by having the same occupational therapist collect AMPS data across sessions, eliminating concerns related to inter-rater reliability [43]. However, the combination of SCED and AMPS also introduced methodological considerations, as the requirement for repeated, comparable measurements within SCED [29] conflicted with the use of varied and context-dependent tasks in AMPS [40]. In addition to these considerations, the inclusion of self-reported outcomes based on AMPS terminology and concepts [40] provided insight into perceived effort, efficiency, safety, satisfaction, and independence. However, as these outcomes were study-specific, their reliability and validity remain uncertain, and the results may be influenced by response bias, including ceiling effects [65] and social desirability bias [73].

Building on the transactional perspective of AT [7], and in line with previous methodological discussions [28–30], we consider SCEDs methodologically applicable and relevant to explore AT efficacy, as supported by the findings of the present study. By incorporating repeated measures and allowing each participant to serve as their own control, SCED accommodates the inherently individual nature of AT use by capturing not only variability between participants but also within-participant variability across situations [29]. Another strength of SCED is its suitability for heterogeneous samples, which are common in AT [24] and evident in the present study. However, although activity chair provision in Denmark appears to follow a gendered pattern, with women comprising the majority of users, approximately 20% of users in the previous Danish study were men [9]. The exclusively female sample in the present study therefore limits representation of the broader user population, particularly as gender has been shown to influence AT-related outcomes [74]. Regarding sample size, the six participants included in this study represent a relatively large sample within a SCED context [43]. However, the sample remains small compared with group-based designs, and concerns regarding generalisability are therefore commonly raised in relation to SCEDs [75]. Yet, larger sample sizes in group-based designs do not in themselves ensure more generalisable findings, as analyses based on group means may mask individual responses [4, 8, 9]. Generality is therefore ultimately established through replication across participants, settings, and contexts, rather than by design alone [75].

While focusing on the activity chair allowed us to explore the efficacy of one specific type of AT, the findings may not be generalisable to other types of AT, which serve different purposes and influence occupational performance in different ways. Further research is needed to examine the efficacy of other types of AT across more diverse samples, including greater gender diversity, and explore the applicability of different SCED designs in AT research.

## Conclusion

This study demonstrated that the efficacy of the activity chair in terms of quality of occupational performance varied across and within participants. No consistent improvements in overall motor and process skills were identified. At the level of specific performance skills, however, the activity chair was associated with higher ratings for bending, reaching, lifting, and transporting objects, but lower ratings for positioning and navigation. Several participants reported reduced effort when using the activity chair, while task completion time generally increased. Together, these findings support a transactional understanding of AT, where efficacy is not inherent but emerges through transactions between the user, the occupation, the AT, and the environment. The findings further highlight the importance of evaluating AT in specific situations, combining observed and self-reported outcomes, and considering the provision process when interpreting AT outcomes.

## Data Availability

Data are available from the corresponding author upon reasonable request and subject to institutional approval.
